# Unlocking Single‐Particle Multiparametric Sensing: Decoupling Temperature and Viscosity Readouts through Upconverting Polarized Spectroscopy

**DOI:** 10.1002/smtd.202400718

**Published:** 2024-11-03

**Authors:** Elisa Ortiz‐Rivero, Katarzyna Prorok, Riccardo Marin, Artur Bednarkiewicz, Daniel Jaque, Patricia Haro‐González

**Affiliations:** ^1^ Nanomaterials for Bioimaging Group, Departamento de Física de Materiales, Facultad de Ciencias Universidad Autónoma de Madrid Madrid 28049 Spain; ^2^ Instituto de Materiales Nicolás Cabrera Universidad Autónoma de Madrid Madrid 28049 Spain; ^3^ Institute of Low Temperature and Structure Research Polish Academy of Sciences Okolna 2 Wroclaw 50‐422 Poland; ^4^ Institute for Advanced Research in Chemical Sciences (IAdChem) Universidad Autónoma de Madrid Madrid 28049 Spain

**Keywords:** optical trapping, rheometer, single‐particle thermometer, upconverting particles

## Abstract

Upconverting particles (UCPs), renowned for their capability to convert infrared to visible light, serve as invaluable imaging probes. Furthermore, their responsiveness to diverse external stimuli holds promise for leveraging UCPs as remote multiparametric sensors, capable of characterizing medium properties in a single assessment. However, the utility of UCPs in multiparametric sensing is impeded by crosstalk, wherein distinct external stimuli induce identical alterations in UCP luminescence, hindering accurate interpretation, and yielding erroneous outputs. Overcoming crosstalk requires alternative strategies in upconverting luminescence analysis. In this study, it is shown how a single spinning NaYF_4_:Er^3+^, Yb^3+^ upconverting particle enables simultaneous and independent readings of temperature and viscosity. This is achieved by decoupling thermal and rehological measurements—employing the luminescence of thermally‐coupled energy levels of Er^3+^ ions for thermal sensing, while leveraging the polarization of luminescence from non‐thermally coupled levels of Er^3+^ ions to determine viscosity. Through simple proof‐of‐concept experiments, the study validates the capability of a single spinning UCP to perform unbiased, simultaneous temperature, and viscosity sensing, thereby opening new avenues for advanced sensing in microenvironments.

## Graphical abstract

Upconverting particles (UCPs) are valuable imaging probes due to their ability to convert infrared to visible light and respond to various stimuli. However, crosstalk among stimuli limits their multiparametric sensing utility. This study introduces a NaYF_4_:Er^3+^, Yb^3+^ UCP that enables simultaneous, reliable temperature, and viscosity readings by separating thermal and mechanical measurements, offering promising advancements in sensing applications.

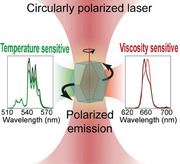

## 1 Introduction

In biological systems, the interplay between temperature and viscosity is pivotal. For instance, fluctuations in intracellular viscosity can arise from alterations in cytoskeletal structure or changes in local temperature: two phenomena occurring concomitantly during cell development.^[^ ^1^, ^2^ ^]^ Hence, a comprehensive understanding of the dynamics within biological microenvironments requires that both temperature and viscosity are monitored simultaneously and in a remote way, thus minimizing perturbations to the investigated system (e.g., a cell).

Individual luminescent particles have emerged as prime probes for remote and high‐resolution sensing, offering unprecedented insights into the behaviors of binary mixtures,^[^ ^3^ ^]^ chemical reactions, complex liquids,^[^ ^4^ ^]^ and cytoplasm.^[^ ^5^ ^]^ Among these luminescent particles, erbium‐doped upconverting particles (UCPs) stand out due to their sensitivity to temperature,^[^ ^6^, ^7^, ^8^, ^9^, ^10^ ^]^ and mechanical pressure.^[^ ^11^, ^12^, ^13^ ^]^ Notably, the temperature‐sensing capability of erbium‐doped UCPs is well established, relying on spectral analysis of their green emission stemming from two thermally coupled states.^[^ ^14^, ^15^, ^16^, ^17^, ^18^ ^]^ Furthermore, UCPs can be utilized for remote viscosity sensing through the analysis of the rotation dynamics of a single UCP within an optical trap.^[^ ^19^, ^20^ ^]^ Rotation dynamics can be extracted from examining the fluctuation in the polarization state of upconverting luminescence and its correlation with particle orientation.^[^ ^21^, ^22^ ^]^ Consequently, the analysis of the luminescence from a single UCP holds promise for simultaneous determination of viscosity and temperature. Previous experiments using UCPs for intracellular sensing have separately reported on temperature or viscosity, but never on the simultaneous readout of these two parameters.^[^ ^23^ ^]^ This is because of challenges posed by crosstalk: a phenomenon where different external stimuli – herein temperature and viscosity – induce similar or indistinguishable changes in luminescence, thus yielding faulty readouts.^[^ ^24^ ^]^ A strategy to effectively separate the response to temperature and medium viscosity via luminescence sensing is thus required to achieve accurate readouts of these parameters of great biological relevance.

This study showcases the feasibility of decoupling temperature and viscosity readouts through multiband analysis of luminescence from a single UCP. We present reliable, crosstalk‐free multiparametric sensing using a single UCP rotating within an optical trap, leveraging analysis of its visible anti‐Stokes emission spectrum. The green luminescence from thermally‐coupled states of Er^3+^ ions provides the thermal readout, while real‐time analysis of red luminescence polarization enables determination of spinning speed and, consequently, medium viscosity.

## 2 Results and Discussion

### 2.1 Single Particle Polarized Spectroscopy

**Figure 1:**
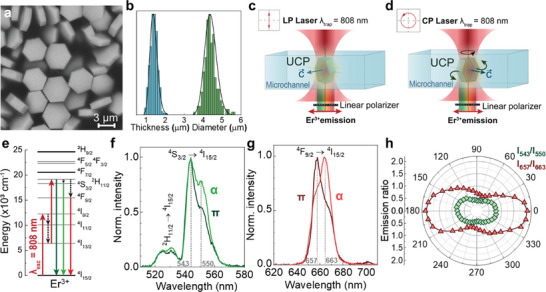
a) SEM image of the NaYF_4_: Er^3+^, Yb^3+^ upconverting microparticles used in this work. b) Normalized size histograms obtained from the analysis of SEM pictures (over 200 particles were measured), with black lines representing lognormal fits. Schematic diagram to describe the orientation of a UCP within an optical trap when the trapping beam is linearly polarized (c) or circularly polarized (d). e) Energy levels diagram of Er^3+^ ions in NaYF_4_ indicating the different excitation and emission processes (solid lines) and multi‐phonon relaxation (dashed lines) of relevance in this work. Normalized f) “green” and g) “red” emission spectra of a single UCP, corresponding to the two orthogonal α (light curves) and π (dark curves) configurations, i.e., light polarized perpendicular and parallel to the optical axis $\vec{c}$ of the UCP, respectively. Dashed lines indicate the two wavelengths (for each band) where the polarization dependence of luminescence becomes more evident. h) Polar plots of the intensity ratios calculated at the wavelengths indicated by dashed lines in (f) and (g) (green and red points, respectively).

Birefringent upconverting NaYF_4_ microparticles doped with Er^3+^ and Yb^+3^ ions were synthesized by a hydrothermal method, as described elsewhere.^[^ ^25^ ^]^ These particles present a hexagonal disk‐shaped morphology (see **Figure** **1**a) with average thickness and diameter of 1.4 ± 0.2 µm and 4.4 ± 0.3 µm, respectively (see Figure 1b and data included in Synthesis section of Experimental Section). The hexagonal β‐phase crystal structure of the UCPs was confirmed by X‐ray diffraction analysis (see Section S1 and Figure S1, Supporting Information) A single‐beam optical tweezers setup is used for the isolation of a single UCP. Optical trapping is achieved by focusing an 808 nm laser beam into the chamber containing an aqueous UCP suspension. The optical torque orientates the UCP within the trap with its hexagonal face parallel to the wavevector of the 808 nm radiation. If the 808 nm laser radiation is linearly polarized, then the UCP reaches a fixed position with its hexagonal face parallel to the laser polarization (Figure 1c). On the other hand, when the 808 nm laser radiation is circularly polarized, the transfer of angular momentum results in a continuous rotation of the UCP (Figure 1d).^[^ ^26^ ^]^ The polar plots of the 808 nm trapping radiation when it is linearly and circularly polarized are included in Figure S2 (Supporting Information).

The presence of both Er^3+^ and Yb^3+^ ions within the UCP allows optical excitation of visible emission under either 808 or 980 nm illumination. Due to the water absorption band at 980 nm and to avoid undesirable laser heating, in this work experiments were performed under 808 nm excitation.^[^ ^27^ ^]^ Under direct Er^3+^ excitation at 808 nm, the Er^3+^ ions in the NaYF_4_ crystal are directly excited up to high energy levels via sequential absorption of pump photons (Figure 1e), thus resulting in a visible emission from Er^3+^ ions. This visible emission is constituted by three bands, centered at 530, 550, and 660 nm, corresponding to the ^2^H_11/2_,^4^S_3/2_ → ^4^I_15/2_ (green bands) and to the ^4^F_9/2_ → ^4^I_15/2_ (red band) transitions, respectively.^[^ ^28^, ^29^, ^30^ ^]^ Because of the anisotropic crystal field of NaYF_4_ UCPs, both the green and red bands are polarized, and the shape of the emission spectra depends on the relative orientation between the electric field of the emitted radiation ($\vec{E_{em}}$) and the optical axis of the NaYF_4_ UCP ($\vec{c}$ axis, that aligns perpendicularly to the hexagonal face of the UCP).^[^ ^22^ ^]^ To assess the polarized emission of an individual UCP in water, a single UCP was optically trapped with a linearly polarized 808 nm laser beam that resulted in its fixed orientation within the trap. The polarization properties of the visible emission were recorded using a rotating linear polarizer located in the emission pathway. Figure 1f,g show the green and red emission spectra from a single, optically trapped, NaYF_4_ UCP as obtained when $\vec{E_{em}}⊥\vec{c}$ (α configuration) and when $\vec{E_{em}}∥\vec{c}$ (π configuration). The dashed lines in Figure 1f,g indicate the specific wavelengths where the polarization dependence of luminescence becomes more evident. The obtained polar diagrams of the ratio between these intensities (Figure 1h) reveal that both green and red bands are polarized, although the red band exhibits a significantly larger degree of polarization dependence. Indeed, the degree of polarization calculated for the I_543_/I_550_ (green) and I_667_/I_663_ (red) intensity ratios were determined to be 0.28 and 0.44, respectively.

### 2.2 Single Particle Polarized Luminescence Thermometry

**Figure 2:**
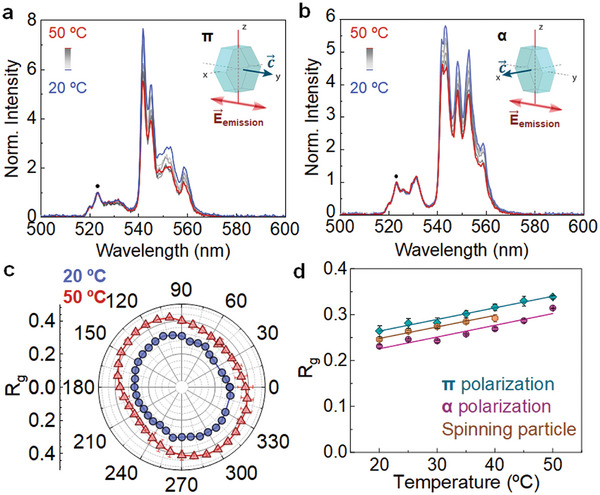
a,b) Normalized green emission bands generated by a single NaYF_4_:Er^3+^, Yb^3+^ UCP excited at 808 nm, as obtained for different temperatures for the two polarization eigenstates (π and α, respectively). c) Angular plot of the green intensity ratio (*R_g_*, as defined in Equation (1)) as obtained from a single, non‐rotating UCP at 20 and 50 °C. d) Temperature dependence of *R_g_* as obtained for the two polarization eigenstates (α and π) and for a rotating particle. Data obtained from the analysis of the upconverting luminescence generated from a single, non‐rotating UCP and from a single, rotating UCP. Error bars were calculated from the analysis of consecutive spectra and taking into account the signal noise in our measurements.

The two excited states involved in the green emission (^2^H_11/2_ and ^4^S_3/2_) are thermally coupled so that their populations follow a Boltzmann distribution.^[^ ^31^ ^]^ In these conditions the luminescence intensities associated to the ^2^H_11/2_ → ^4^I_15/2_ transition (*I_H_*) and to the ^4^S_3/2_ → ^4^I_15/2_ transition (*I_S_*) of Er^3+^ ions are not independent but connected through temperature:^[^ ^32^ ^]^

(1) $$R_{g}=\frac{I_{H}}{I_{S}}=C\exp\left(-\frac{\Delta E}{k_{B}T}\right)$$

where *R_g_* is the intensity ratio between the green emission bands, *I_H_* and *I_S_* are the integrated intensities of the ^2^H_11/2_ → ^4^I_15/2_ and ^4^S_3/2_ → ^4^I_15/2_ transitions, respectively; *C* is a constant that depends on the degeneracy of the emitting levels, spontaneous emission rate, and photon energies of the emitting states in the host materials; Δ*E* is the energy gap separating the two excited states, *k_B_* is the Boltzmann constant, and *T* is the absolute temperature. Hereafter both *I_H_* and *I_S_* intensities are calculated by integrating the emitting spectra in specific wavelength ranges ($I_{H}=\int_{515}^{535\;}I_{em}\left(\lambda \right)d\lambda$ and $I_{S}=\int_{535}^{550}I_{em}\left(\lambda \right)d\lambda$) to avoid considering signal coming from transitions involving levels not thermally coupled.^[^ ^33^ ^]^ Equation (1) reveals that temperature determines the spectral shape of the green band (constituted by the ^2^H_11/2_ → ^4^I_15/2_ and ^4^S_3/2_ → ^4^I_15/2_ transitions). From the emission spectra obtained at different temperatures (**Figure** **2**a,b), it is experimentally observed that the relative contribution of the ^4^S_3/2_ →^4^I_15/2_ emission decreases with temperature for both polarizations, as predicted by Equation (1). This temperature‐induced change in the *R_g_* intensity ratio has been widely used for remote thermal sensing.^[^ ^23^ ^]^ Indeed, Er^3+^‐luminescence‐based thermal sensing using UCPs requires the measurement of *R_g_*, which is converted into temperature by using a previously acquired *R_g_* versus *T* calibration curve.^[^ ^34^, ^35^, ^36^ ^]^ This approach has been widely applied to isotropic UCPs that produce non‐polarized luminescence. But in our case, the situation requires a deeper analysis. As described in Section 2.1, the shape of the green bands also depends on the polarization state of the emitted radiation (Figure 1). This means that changes in *R_g_* could not be unequivocally ascribed to changes in the UCP temperature, as *R_g_* could be also affected by changes in its orientation. We measured the polarization dependence of *R_g_* for a fixed UCP (a single UCP optically trapped with a linearly polarized laser beam, Figure 2c) at 20 and 50 °C. Experimental data reveals that, independently of the polarization of the emitted radiation, *R_g_* increases with temperature (as predicted by Equation (1)). At the same time, *R_g_* is not fully isotropic as it depends on the polarization of the emitted radiation (Figure 2c). The data included in Figure 2c reveals a degree of polarization for *R_g_* close to 0.1 (i.e., *R_g_* is 90% unpolarized). This means that *R_g_* is not a valid thermometric parameter as it also depends on the particle orientation. To make *R_g_* a reliable thermometric parameter it should be averaged during a complete rotation. This can be done by either setting an acquisition time longer than the rotating period or by averaging emission spectra acquired during a complete rotation. In this case, the calibration curve to be used for thermal sensing should be acquired from a rotating particle using spectra averaged, at least, over a whole rotation period. Figure 2d includes the calibration curves (*R_g_* vs T) obtained from a static UCP for the two polarization eigenstates (α and π). Due to the anisotropy revealed by the polar diagrams (Figure 2c), the calibration curves are different for these two polarizations. When the calibration curve is measured for a rotating UCP the averaged *R_g_* lies between the values obtained for α and π polarizations, as expected (Figure 2d). The thermal sensitivities calculated from data included in Figure 2d are included in Figure S3 (Supporting Information). It should be noted that the determination of the temperature of the UCP from the analysis of *R_g_* requires the use of an integration (acquisition) time larger than the thermalization time of the thermally coupled states that, as explained in Section S4 (Supporting Information), has been determined to be close to 50 µs. As it is described later, integration times used all along this work are >20 ms, i.e., two orders of magnitude longer than the thermalization time of thermally coupled states.

### 2.3 Luminescence‐Based Velocimetry for Viscosity Sensing

**Figure 3:**
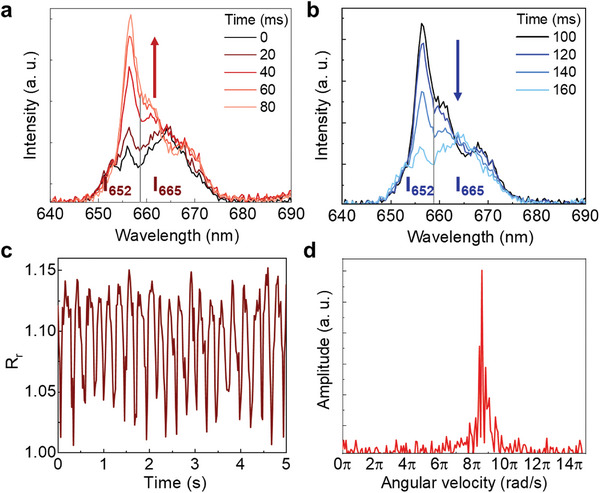
a,b) Red (^4^F_9/2_ → ^4^I_15/2_) emission generated by a single UCP as recorded at a rate of 50 Hz during rotation (integration time for each spectrum was 20 ms, 50 spectra were acquired per second). The rotation of the optical axis changes the polarization of the detected emission and, hence, the shape of the red emission band. c) Time‐evolution of the intensity ratio  *R_r_* = *I*_652_/*I*_665_  between the integrated emitted intensities centered at 652 and 665 nm, as defined in main text, as obtained from a UCP rotating with constant angular speed. d) Frequency‐domain spectrum of the red ratio fluctuations, which gives the angular speed of the spinning microparticle.

When the 808 nm trapping radiation is circularly polarized, the light‐to‐particle transfer of momentum triggers the rotation of the UCP within the optical trap, as previously described.^[^ ^37^, ^38^ ^]^ The steady‐state angular speed of a single NaYF_4_ UCP is given by:

(2) $$\Omega =\alpha \frac{P}{\eta }$$

where α is a constant that depends on the geometry and birefringence of the UCP, *P* is the trapping laser power, and η is the medium viscosity. Therefore, the determination of the angular speed of an individual NaYF_4_: Er^3+^, Yb^3+^ UCP rotating within an optical trap can be used for remote sensing of medium viscosity.^[^ ^39^, ^40^ ^]^ In previous works the spinning speed (Ω) was experimentally determined by using quadrant photodetectors or video processing.^[^ ^41^, ^42^ ^]^ In this work, we propose an alternative approach based on the determination of Ω from the analysis of the polarized upconversion luminescence. Figure 1h reveals that the intensity ratio between emission lines within the red band strongly depends on the polarization of emitted radiation. If the polarization of the detected emission is fixed, then the intensity ratio  *R_r_* = *I*_652_/*I*_665_  between the integrated intensities $I_{652}=\int_{645}^{659\;}I_{em}\left(\lambda \right)d\lambda$ and $\;I_{665}=\int_{659}^{680}I_{em}\left(\lambda \right)d\lambda$ will fluctuate periodically with time (**Figure** **3**a–c) with a characteristic frequency Ω. In this way the rotation speed of the UCP (Ω) can be determined from the Fourier analysis of *R_r_* (*t*) (Figure 3d). Once the rotation speed is determined, the viscosity is given by:

(3) $$\eta =\alpha \frac{P}{\Omega }$$

It is important to remark here that the measured viscosity corresponds to the viscosity of the medium in physical contact with the rotating UCP, i.e., at the local temperature *T_l_* that is here assumed to be equal to the temperature of the UCP. We have also explored the possible temperature dependence of *R_r_* (see Section S5 and Figure S4, Supporting Information) concluding that it is, within experimental error, a temperature‐independent intensity ratio. This was, indeed, expected as this red band is not generated by thermally coupled states. Thus, we conclude how a proper analysis of the shape fluctuations of the red band provides us with a reliable reading of the rotation speed.

### 2.4 Real‐Time, Decoupled Temperature and Viscosity Sensing

**Figure 4:**
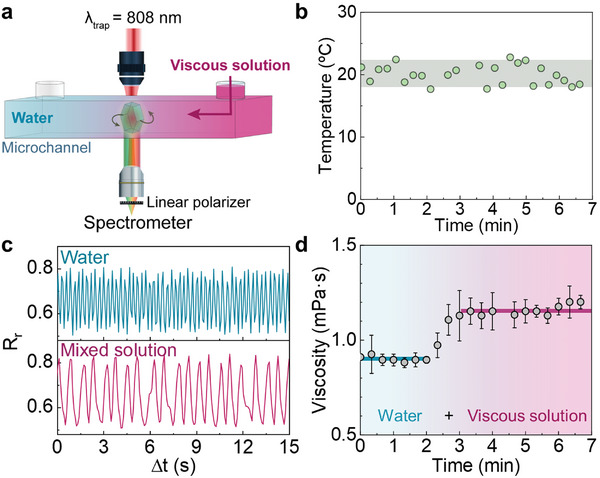
a) Schematic representation of a trapped and rotated particle with a circularly polarized 808 nm laser at constant power of 105 mW (power density of 6.8 MW cm^−2^) in a microchannel filled with water. At a certain moment, a solution of 1.88 mg mL^−1^ of polyacrylamide (PAM) in water is introduced through one of its inlets. b) Time evolution of local temperature as determined from the analysis of *R_g_*. Dots are experimental data, and shaded area indicates the standard deviation of measurements. c) Time fluctuation of the intensity ratio *R_r_* before (blue) and after (pink) the viscous medium has diffused within the chamber. Integration time was 100 ms, ensuring the acquisition of up to 6 data per complete rotation of the UCP. d) Time evolution of the local viscosity as obtained from the Fourier analysis of the time fluctuation of *R_r_*. Dots are experimental data, and horizontal lines indicate the local viscosity before and after the diffusion of the viscous medium.

Sections 2.2 and 2.3 revealed how multiband ratiometric analysis of the luminescence generated by a rotating UCP opens the possibility of decoupled readout of temperature and viscosity. To demonstrate this, we conducted a proof‐of‐concept experiment in which the rotating UCP is used for simultaneous sensing of viscosity and temperature during fluid mixing (**Figure** **4**a). A single UCP is optically trapped and rotated within a microchamber by a circularly polarized 808 nm laser beam. The microchamber is placed on a stage at a constant temperature of 20 °C. The absence of any relevant laser‐induced heating has been confirmed from the analysis of both the Er^3+^ luminescence and the rotation dynamics, as described in Section S6 (Supporting Information) (Figure S5, Supporting Information). Initially, the microchamber was filled with water. At a specific timepoint, a second liquid – consisting of a viscous solution of polyacrylamide (PAM) in water with a viscosity of 1.88 mPa·s also at a temperature of 20 °C – was injected into the chamber through one of its inlets. Details about the injection procedure and solution properties can be found in the Experimental Section, and the emission spectra of the rotating UCP is shown in Section S7 (Supporting Information) (Figure S6, Supporting Information). Time evolution of local temperature and viscosity before, during, and after the diffusion of the viscous solution was determined from the ratiometric analysis of both green and red bands, respectively, as explained in Sections 2.2 and 2.3. The time evolution of the local temperature (Figure 4b) reveals an average temperature of 19.9 °C, in very good agreement with the temperature set externally in the microchamber (20 °C). Statistical analysis of experimental data reveals a temperature uncertainty, estimated from the standard deviation of temperature readouts, of ±1.5 °C. Note that the diffusion of the viscous PAM solution does not lead to any noticeable change in the temperature sensed by the particle within the abovementioned uncertainty. This was, indeed, expected as the injected viscous medium was at the same temperature as the water initially filling the microchamber. Figure 4b reveals that any change in local viscosity cannot be correlated with temperature and, thus, should be caused by the diffusion of the viscous medium through the chamber. Figure 4c includes the time fluctuation of the intensity ratio *R_r_* before (blue) and after (pink) the viscous medium has fully mixed with the water within the chamber. When the viscous liquid diffuses along the chamber there is a slow‐down of the particle spinning, indicating an increment in local viscosity. The time evolution of local viscosity was determined from the Fourier analysis of the *R_r_* versus time (Figure 4d) at different time points after the injection of viscous liquid. This analysis showed an initial viscosity between 0.9 and 1.0 mPa·s (expected for pure water), followed by a rapid increase to an average value of 1.2 mPa·s upon intermixing of water with the PAM solution. As expected, the latter value is lower than the one of the PAM solution (1.88 mPa·s), due to the dilution of the polymer solution in pure water.

**Figure 5:**
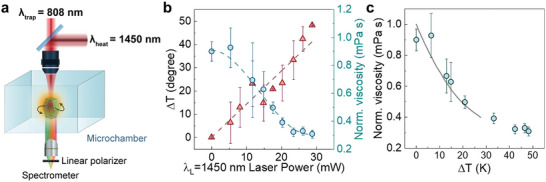
a) Schematic representation of a trapped and rotated particle with a circularly polarized 808 nm laser in the presence of a 1450 nm laser beam used for local heating. The medium filling the microchamber is an aqueous solution of polyacrylamide. b) Local temperature increment (triangles) and viscosity change (circles) as obtained for different 1450 nm laser powers. c) Temperature dependence of the viscosity reduction as obtained from the data included in (b) (circles). The results previously published by using a viscometer^[^ ^43^ ^]^ are also included as a solid line.

Once the ability of a rotating UCP for decoupled measurement of temperature and viscosity has been demonstrated, we applied this method to determine the temperature dependence of viscosity of an aqueous solution of PAM using the set‐up represented in **Figure** **5**a. An individual UCP was optically trapped and rotated within a microchamber by using a focused 808 nm laser beam. The microchamber was filled with a polyacrylamide (PAM) solution whose viscosity versus temperature curve is unknown. The (negligible) effect of PAM on the spectroscopic properties of our UCP are discussed in Supporting Information (Section S8 and Figure S7, Supporting Information). The polarized luminescence generated by this UCP was registered to achieve simultaneous reading of both temperature and viscosity. Local temperature was varied by using an additional single‐mode diode laser operating at 1450 nm (radiation highly absorbed by water) that is coupled to the experimental system and that overlaps with the 808 nm trapping laser. The laser beam radius at the focus of the 808 and 1450 nm radiation within the chamber were estimated to be 0.7 and 1.1 µm, respectively. The power of the 808 nm laser was kept constant (70 mW, corresponding to 4.5 MW cm^−2^) during the experiment to ensure a constant optical torque. On the other hand, the 1450 nm laser power was varied between 0 and 30 mW (from 0 to 0.8 MW cm^−2^) to change the local temperature of the fluid. For each value of the 1450 nm laser power, we analyzed the green and red bands luminescence to determine the local temperature and viscosity, respectively (see Figure 5b). As expected, and in agreement with previous works, the local temperature increases linearly with the 1450 nm laser power while the local viscosity decreases due to the local heating. Combining the data shown in Figure 5b, we obtained the temperature‐induced reduction in the local viscosity of the polyacrylamide solution (Figure 5c). The experimental data can be compared with previous reports on the temperature dependence of the viscosity of similar solutions.^[^ ^43^ ^]^ A reasonably good agreement between our data and those obtained by rheological measurements using a viscometer (solid line in Figure 5c) is observed, which clearly confirms the validity of our approach for simultaneous measurement of the temperature and the viscosity.

## 3 Conclusion

In summary, in this work, we have introduced a novel strategy to achieve uncoupled and simultaneous readouts of local viscosity and temperature. The readout of these two parameters, unaffected by cross‐talk, is achieved by analyzing the luminescence generated by an optically trapped NaYF_4_: Er^3+^, Yb^3+^ rotating microparticle. Real‐time analysis of the green emission of erbium ions, generated by thermally coupled levels, provides the thermal readout. At the same time, the highly polarized red emission of erbium ions allows extracting the UCP rotating speed, from which the local viscosity is determined. Since temperature and viscosity readouts are based on different physical phenomena and are obtained from the analysis of different emission bands, they are decoupled. The ability of the NaYF_4_: Er^3+^, Yb^3+^ rotating microparticle to provide reliable, simultaneous, and decoupled readouts of viscosity and temperature has been used in two proof‐of‐concept experiments. Therein, we were able to individually monitor temperature and viscosity during the mixing of two liquids and during local heating of a liquid medium.

The results presented here pave the way for the development of fast and miniaturized systems for local characterization of biological media at the microscale that could overcome the current limitations of luminescent sensors. For example, this study could enable further analysis of different biosystems or temperature‐changing events such as in situ monitoring of reaction processes and other complex mechanisms.

## 4 Experimental Section

### Synthesis

Yttrium oxide (99.99%), ytterbium oxide (99.99%), erbium oxide (99.99%), and sodium fluoride were purchased from ALDRICH Chemistry. Lanthanide nitrates were obtained by reacting a stoichiometric amount of lanthanide oxides with nitric acid. Ethanol (96% pure p.a.), sodium citrate (pure p.a.), and nitric acid (pure p.a.) were purchased from Avantor (Poland).

The NaYF_4_ microparticles doped with 20 mol.% Yb^3+^ and 2 mol.% Er^3+^ ions were prepared using the hydrothermal method. Aqueous solutions of sodium citrate (9.31 mL; 0.3 m) and Ln(NO_3_)_3_ (14 mL; 0.2 m; Ln = Y, Er) were mixed under vigorous stirring to form a milky suspension. Then an aqueous solution of NaF (44.8 mL; 0.5 m) was added to the beaker to form a transparent solution. The mixture was transferred to a 100‐mL Teflon vessel and heated to 220 °C for 12 h. After being cooled to room temperature, the reaction product was isolated by centrifugation and washed with ethanol. Finally, the prepared particles were dispersed in water.

### Setup Design

**Figure 6:**
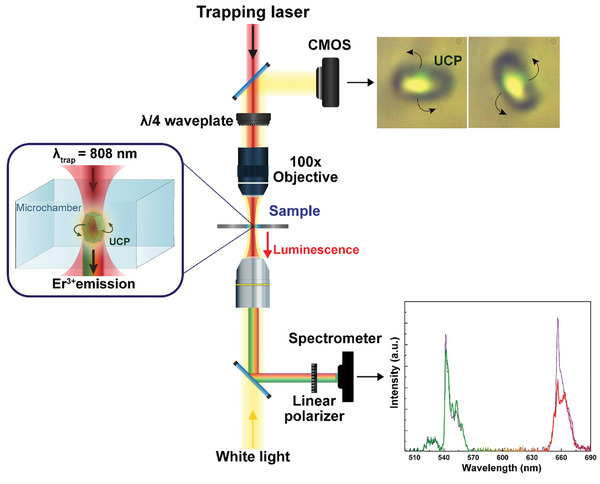
Schematic representation of the optical trapping setup.

Single NaYF_4_ microparticles were optically trapped using a homemade single‐beam optical tweezers setup. A linearly‐polarized single‐mode fiber‐coupled 808‐nm laser diode was used as laser radiation. The optical system is schematically represented in **Figures** **1**, **6**.

A drop of a colloid of UCPs in water, previously stirred to avoid clusters, was pipetted into a IBIDI micro‐chamber and placed in the optical tweezers' setup. The light beam provided by the diode laser was focused into the chamber containing the UCPs by using a LCPLN 100x IR Olympus microscope objective with a Numerical Aperture of 0.85. To induce the UCPs’ rotation, a quarter‐wave plate was placed before the focusing objective to convert the linearly polarized light into circularly polarized light by inducing a relative phase shift Δφ of 90°. In these experimental conditions, a single UCP can be simultaneously optically trapped, excited, and rotated. Real time optical imaging of the particle was achieved by coupling a white LED, focused on the sample by an objective lens with low numerical aperture, and using a CMOS camera incorporated into the system together with a spectral filter used to remove the laser light. In Figure 6 two optical images are shown, where the upconversion luminescence of the trapped UCP can be observed. The lower objective lens is used as a light condenser, but it also serves as a collector lens to focus the UC luminescence into a costume‐made Ocean Optics QE65000 High‐Sensitivity Fiber Optic Spectrometer, with a wavelength range of detection of 400–700 nm and a spectral resolution of one nanometer. Therefore, the upper part of the experimental setup allows the optical trapping of a single microparticle and its rotation, while the lower section of the setup allows the detection of the luminescent particle.

### Injection Procedure

A colloidal of UCPs in water was introduced in an IBIDI µ‐Slide microchamber and a single UCP was trapped and rotated. Then a solution of 1.88 mg·mL^−1^ of polyacrylamide in water was injected in the microchamber by one of its inlets. This solution was mixed with 0.3 mg mL^−1^ of Rhodamine B, a tracer dye that allows the visualization of the liquid flow inside the microchamber. The presence of Rhodamine B, although its absorption overlaps with the erbium emission, does not hinder the reliability to the thermal measurements as explained in Section S9 (Supporting Information) (Figure S8, Supporting Information).

## Conflict of Interest

The authors declare no conflict of interest.

## Supporting information



Supporting Information

## Data Availability

The data that support the findings of this study are available from the corresponding author upon reasonable request.
