# Supplementary material for: Unlocking Single‐Particle Multiparametric Sensing: Decoupling Temperature and Viscosity Readouts through Upconverting Polarized Spectroscopy
Source: Small Methods. 2024 Nov 3;9(4):2400718. doi: 10.1002/smtd.202400718 (PMC12020337; doi:10.1002/smtd.202400718)
Supplement: Supplementary file 1 — Supporting Information [file SMTD-9-2400718-s001.docx]

Supporting Information

Unlocking Single-Particle Multiparametric Sensing: Decoupling Temperature and Viscosity readouts through Upconverting Polarized Spectroscopy

Elisa Ortiz-Rivero, Katarzyna Prorok, Riccardo Marin, Artur Bednarkiewicz, Daniel Jaque, and Patricia Haro-González

**Sections**

1. **X-ray diffraction analysis**
2. **Polarization characterization of the excitation laser**
3. **Relative sensitivity**
4. **Integration vs thermalization time**
5. **Temperature independence of the red emission ratio R_r_**
6. **Analysis of laser-induced heating**
7. **Spectral data corresponding to Figure 4**
8. **Spectral characterization of UCP in presence of PAM**
9. **Reliability of temperature measurments in presence of Rhodamine B**
10. **References**
11. **X-ray diffraction analysis**

The crystal structure and phase of the NaYF_4_:Er^3+^, Yb^3+^ UCPs were determined via X-ray powder diffraction (XRD) analysis. **Figure S1** presents the XRD pattern of a dried concentrated sample, recorded on an X'Pert PRO X-ray diffractometer with a PIXcel ultrafast line detector, focusing mirror, and Soller slits for Cu Kα radiation. The reflections are consistent with the standard card (ICDD #04-011-3581) (also included in Figure S1), corresponding to the hexagonal phase of NaYF_4_.


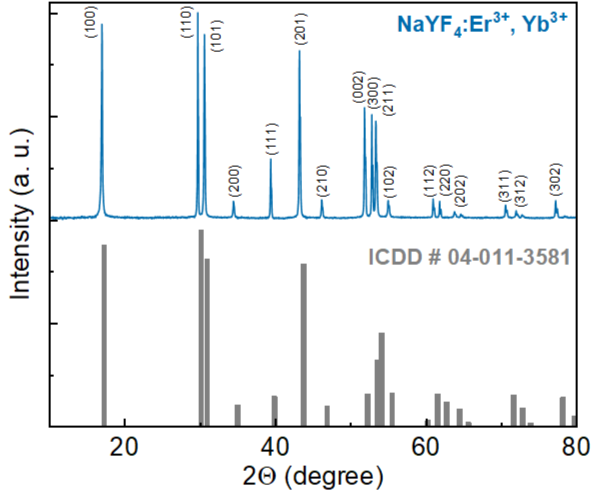


**Figure S1.** XRD pattern of the β-NaYF_4_ UCPs. The standard data of hexagonal NaYF_4_ (ICDD # 04-011-3581) is shown as a reference.

1. **Polarization characterization of the excitation laser**

The 808-nm trapping and excitation laser used in this work is linearly polarized. To induce the rotation of the particle by transfer of spin angular momentum, the laser beam must be circularly polarized. For this reason, a quarter-wave plate was placed after the laser beam in the experimental setup. The polar diagram of the 808-nm trapping radiation has been measured in the presence and absence of the waveplate. In the absence of the waveplate, it is confirmed that the 808 nm trapping radiation is linearly polarized (see polar diagram in **Figure S2a**). For comparison purposes, **Figure S2b** shows the polar diagram of the 808 nm trapping laser when the waveplate is used. The polarization degree of the trapping beam can be calculated from these polar diagrams as follows:

$P=\frac{I_{max}-I_{min}}{I_{max}+I_{min}}$ (S1)

Accordingly, we have obtained a degree of polarization of 0.83 and 0.09 for linear and circular polarization, respectively.


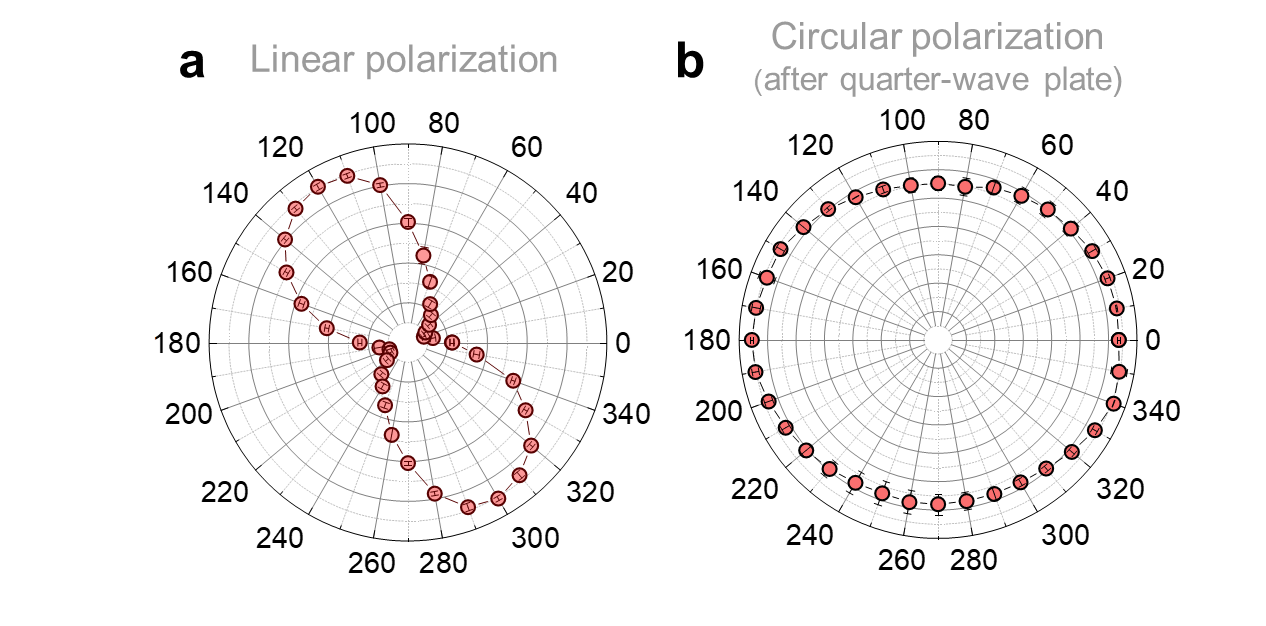


**Figure S2.** Polar plots of the 808 nm trapping radiation as obtained in absence (**a**) and presence (**b**) of the quarter-wave plate corresponding to linear and circular polarization, respectively.

1. **Relative sensitivity**

The relative sensitivity of the UCP can be expressed as:[1]

$S_{r\left( T \right)}=\frac{1}{R_{g}}\frac{dR_{g}}{dT}$ (S2)

Where $R_{g}=\frac{I_{H}}{I_{S}}$ is the luminescence ratio between the green emission bands, $I_{H}$ and $I_{S}$ are the integrated intensities of the ^2^H_11/2_ → ^4^I_15/2_ and ^4^S_3/2_ → ^4^I_15/2_ transitions, respectively, from the thermally coupled energy levels of Er^3+^ ions.


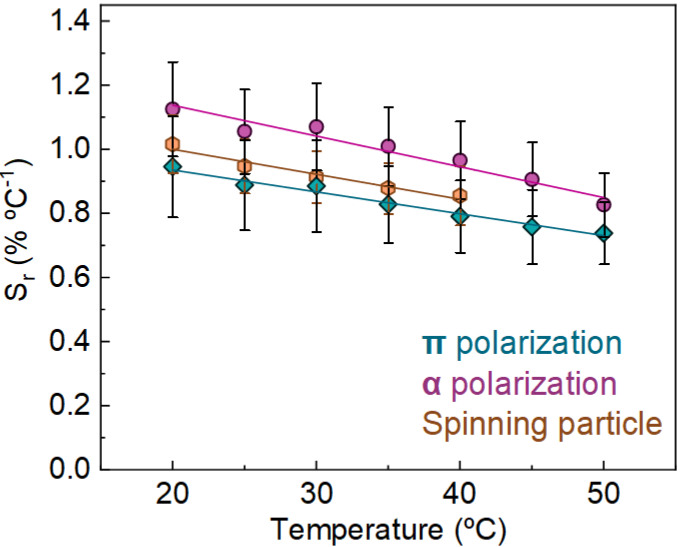


**Figure S3.** Relative sensitivity as a function of temperature for a spinning (orange hexagons) and non-spinning (green squares) luminescent UCP suspended in water.

1. **Integration vs thermalization time**

The two excited states of Er^3+^ ions involved in the green emission (^2^H_11/2_ and ^4^S_3/2_) are thermally coupled so that their populations follow a Boltzmann distribution. In these conditions the luminescence intensities associated to the ^2^H_11/2_ → ^4^I_15/2_ transition ($I_{H}$) to the ^4^S_3/2_ → ^4^I_15/2_ transition ($I_{S}$) of Er^3+^ ions are not independent but connected through temperature. Thus, the R_g_ is temperature-dependant, as shown in the main text.

The integration (acquisition) time of the recorded emission spectra should be larger than the thermalization time of the thermally coupled levels ^2^H_11/2_ and ^4^S_3/2_. The thermalization time of the thermally coupled states ($\tau_{ther}$), in a first order approximation, can be estimated by:

$\tau_{ther}\approx{W_{NR}\left( T \right)}^{-1}$ (S3)

where $W_{NR}\left( T \right)$is the multiphonon (nonradiative) decay rate that is given by:

$W_{NR}\left( T \right)=W_{NR}\left( 0 \right)\left[ 1-exp\left( \frac{-\hbar\omega}{k_{B}T} \right) \right]^{-{\Delta E}/{k_{B}T}}$ (S4)

where $\hbar\omega$ is the energy of the effective phonon mode involved (230 cm^-1^), $k_{B}T$ is the thermal energy (207 cm^-1^), and $\Delta E$ is the energy difference between the thermally coupled states (692 cm^-1^). In equation (**S4),** $W_{NR}\left( 0 \right)$ is the spontaneous phonon emission rates between the thermally coupled levels that is given by the energy gap law:

$W_{NR}\left( 0 \right)=\beta\cdot exp\left( -\alpha\Delta E \right)$ (S5)

where $\beta$ and $\alpha$ are constants that depend on the particular system under study. For the case of NaYF_4_ crystals doped with erbium and ytterbium ions these values have been determined by I.M. Gonçalves and co-workers to be *α* = 2*.*20⋅10^-3^ cm^-1^, and *β* = 23*.*6 ms^-1^. [2] Introducing these values into (S5) leads to $W_{NR}\left( 0 \right)$ = 5.1 ms^-1^. Then, applying (S4) we obtain a multiphonon nonradiative decay rate at room temperature between the two thermally coupled states of $W_{NR}\left( RT \right)$= 19.4 ms^-1^ that leads to a thermalization time of thermally coupled states of 51.4 µs. In our work, the integration (acquisition times) was larger than 20 ms, i.e. more than two orders of magnitude larger than the time required for the thermalization of the thermally coupled levels.

1. **Temperature independence of the red emission ratio R_r_**

**Figure S4** shows the emission spectra corresponding to the red emission band of Er^3+^ ions as obtained for the π and α polarizations at different temperatures in the 20-50 ºC range (4a and 4b, respectively). The temperature dependence of R_r_ as obtained for both polarization is shown in **Figure S4c**.


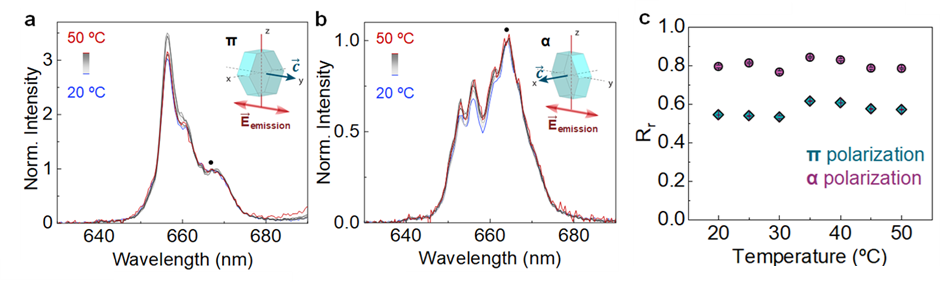


**Figure S4. a,b)** Normalized polarized emission spectra of a single upconverting particle excited at 808 nm, as obtained for different temperatures for the two polarization eigenstates (π and α). **c)** Rr as a function of temperature, obtained for the two polarization eigenstates (α and π). Data obtained from the analysis of the upconverting luminescence generated from a single, non-rotating UCP.

1. **Analysis of laser-induced heating**

The presence of laser-induced heating is a critical factor that could potentially impact the performance of our rotating particle acting as a temperature sensor. When dealing with rotating particles, there is a straightforward way to evaluate the presence of heating: The angular speed (Ω) increases linearly with the trapping laser power in case of no laser-induced heating. On the other hand, if laser radiation locally heats the particle or the liquid medium, the spinning speed increases supra-linearly with the laser power because the temperature of surrounding medium also concurrently increases, which in consequence reduces local viscosity making the particle to rotate faster.

In our experiments we verified that the angular speed followed a linear trend with the increasing applied laser power for the particle rotating in water and in different aqueous solutions of polyacrylamide (see **Figure S5**). Moreover, in these experiments performed at different laser powers, we also determined the temperature of the particle by analysing the spectral shape of the temperature-dependent emission of Er^3+^ ions, which remained constant for the duration of the experiments. Therefore, it is verified that there is no laser-induced heating of the particle.


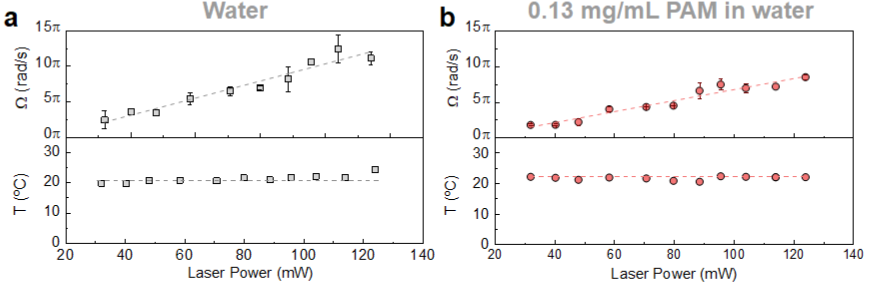


**Figure S5.** Angular speed and temperature of the upconverting particle as a function of the trapping laser power and obtained for different media (water and a solution of polyacrylamide in water). Symbols are experimental data and dashed lines are best linear fits. In both cases, the angular speed increases linearly with the trapping laser power, indicating the absence of relevant local heating. This is supported by the thermal readouts obtained from the analysis of Er^3+^ visible luminescence.

1. **Spectral data corresponding to Figure 4**

We conducted a proof-of-concept experiment in which the rotating UCP is used for simultaneous sensing of viscosity and temperature during fluid mixing (**Figure 4** of the main text). A single UCP is optically trapped and rotated within a microchamber by a circularly polarized 808 nm laser beam. The emission spectra were recorded with a spectrometer coupled to the experimental set-up, and an acquisition time of 100 ms (see **Figure S6**).


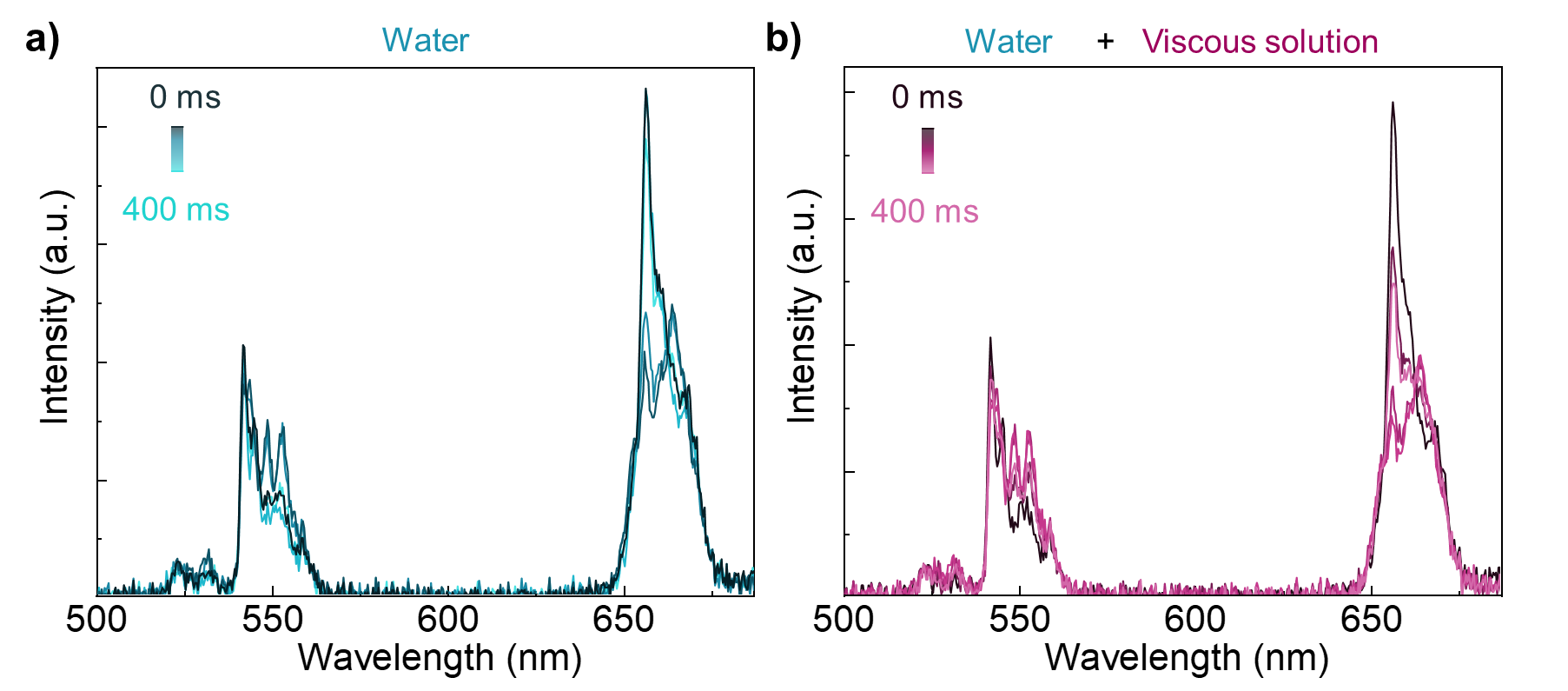


**Figure S6.** Emission spectra corresponding to the trapped and rotating UCP from **Figure 4** of the main text while in **a)** water and in **b)** the aqueous polyacrylamide solution.

1. **Spectral characterization of UCP in presence of PAM**

For temperature and viscosity measurements we are employing intrinsic properties of our UCP. For temperature sensing, we are using the emission generated by two thermally coupled states. For viscosity measurements (based on the determination of rotation speed) we are using the polarized character of the emission bands that, in turn, depends on the crystal symmetry of the host lattice and its impact on the selection rules governing the electronic transitions. So, none of the effects used for sensing are based on or impacted by surface-related properties. The presence of PAM in the surrounding medium can alter the surface-related quenching mechanism and, hence, possibly result in overall lower emitted intensity. This would reduce the signal-to-noise ratio of the signal collected, but it does not bear any effect on the methods used for temperature or viscosity sensing. This reasoning is supported by the data included in **Figure S7**, which show a comparison between emission spectra obtained in presence and absence of PAM. Note that there are differences between the emission spectra of different UCPs even in water (**Figure S7a**) due to intrinsic interparticle variability. When the emission spectra of different particles dispersed in solution with different PAM concentrations are compared, the increasing PAM content does not lead to a systematic variation in the emission spectra (the interparticle differences are dominant over any possible effect of PAM, **Figure S7b**). To support this assessment, we have measured the $R_{g}$ intensity ratio for different particles, laser powers and PAM contents. Results (**Figure S7c**) reveals that, within the experimental uncertainty caused by interparticle differences, the presence of PAM in the medium does not affect $R_{g}$.


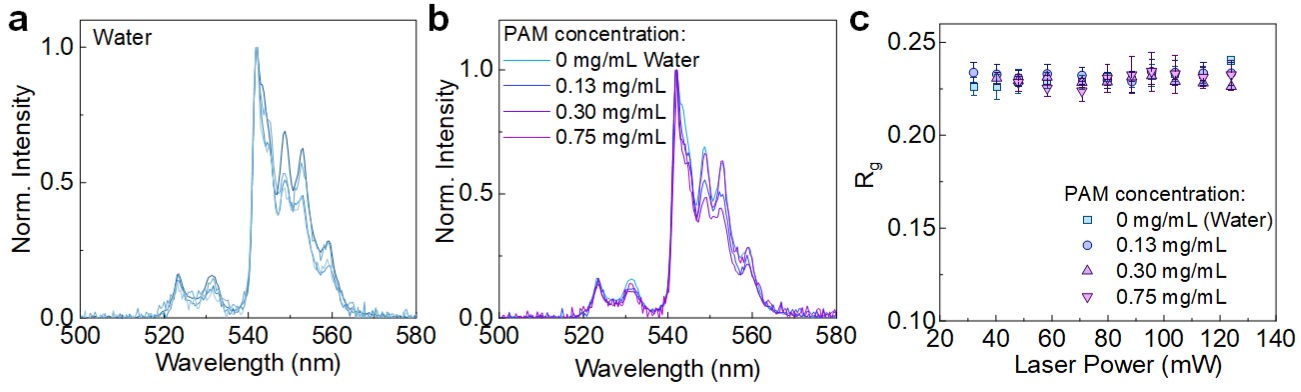


**Figure S7.** Normalized emission spectra of a single upconverting particle excited at 808 nm, as obtained for **a)** different UCPs in water and for **b)** different concentrations of PAM in water. **c)** Green ratio as a function of the applied laser power for rotating particles dispersed in different concentrations of PAM in water. The errors correspond to the standard deviation.

1. **Reliability of temperature measurements in presence of Rhodamine B**

**Figure S8** evidences that a spectral overlap between the absorption of Rhodamine B (RhB) and green emission of erbium ions does exist. The RhB absorption is higher in the 535-575 nm range than in the 515-535 nm range. Thus, the presence of RhB could lead to an increment in the R_g_ intensity ratio used for thermal sensing, and hence to an erroneous thermal reading. In our case we did not detect any relevant spectral distortion due to the presence RhB (see emission spectra from an UCP recorded in presence and absence of RhB included in **Figure S7**). Data of **Figure S7,** indeed, denote a slight increment in the emitted intensity in the 535-575 nm spectral range contrary to the effect expected due to RhB absorption. The analysis of the spectra included in **Figure S7** denotes a decrease of the R_g_ ratio between the two spectra close to ± 1.3% once the RhB has been incorporated into the chamber. This small reduction in the R_g_ ratio can be considered within the experimental error. Independently of the origin of this variation in R_g_, what we can state that it is not due to the presence of RhB (should induce an increment instead of a reduction in R_g_).

As an additional proof that the presence of RhB is not affecting the reliability of our thermal measurements (based on the analysis of R_g_ ratio) is that in the experiments of fluid mixing (**Figure 4** of main text) when the solution containing the RhB reaches the particle we did not detect any sudden change in the shape of the green band (R_g_ remains constant). Note that when the viscous solution reaches the UCP there is a sharp change in viscosity but the temperature (determined from R_g_ ratio) remains unaltered.

The minimum impact of the presence of RhB in our measurements was expected considering the geometry of our experiments. In our experimental conditions, the rotating particle is placed very close to the bottom of the chamber: it is estimated that the particle-bottom distance is 5 microns. The erbium luminescence is collected from the bottom of the chamber, so the luminescence emitted by the particle only passes through a reduced optical path (< 5 µm) of the solution containing the RhB. Thus, the spectral distortion induced by RhB is negligible.

**Figure S8.** Characteristic emission spectra generated from our UCP in the green region as obtained in the presence and absence of RhB within the chamber. The absorption spectrum of RhB is also included to evidence the spectral overlap with the erbium emission.

1. **References**
2. C. D. Brites, *et al.*, *Nanoscale* **2012**, *4*, 4799-4829. DOI 10.1039/C2NR30663H.
3. I. M. Gonçalves, *et al*., *Journal of Luminescence* **2021,** *231*, 117801. DOI 10.1016/j.jlumin.2020.117801.
